# Autistic interests: wide-ranging and intellectually oriented

**DOI:** 10.3389/fpsyt.2026.1749552

**Published:** 2026-08-31

**Authors:** Catherine L. Caldwell-Harris

**Affiliations:** Department of Psychological and Brain Sciences, Boston University, Boston, MA, United States

**Keywords:** academia, autism, careers, discovery, interests, learning, self-regulation

## Abstract

**Introduction:**

The interests of autistic individuals have traditionally been described as circumscribed and restricted, serving the functions of self-regulation by reducing prediction error. Historically, this may have seemed reasonable when applied to autistic children and adults with intellectual disability. The current study investigated the restrictiveness and function of focused interests of autistic adults with typical intellectual ability.

**Methods:**

To hear from autistic adults themselves, bypassing interviewers’ agendas, online discussion posts were analyzed from threads that opened with questions such as *What are your special interests*? Content analysis was utilized to categorize the special interest topics into a taxonomy (e.g., Academic, Entertainment) and to code for functions, including knowledge seeking (systemizing), mastery, self-regulation, and socializing. Word frequency analyses tallied terms associated with intellectual content and intensity, to evaluate whether interests serve knowledge-seeking vs self-regulation.

**Results:**

Academic topics dominated the interests mentioned, appearing in approximately half of all posts, with psychology-related topics being the most frequent, appearing in 29% of posts. Interests were often highly specific, but were frequently the opposite of restricted. Many posts described a large, diverse range of interests, spanning disparate domains, which was labeled *wide-ranging intellectual appetite*. Knowledge seeking emerged as the primary function, with 52% of posts containing relevant terms (e.g., *learn, read, research*). Intellectual approaches to an interest were frequently reported even for non-academic topics like fictional worlds, cooking, pets and true crime. Posts describing the purpose of interests as self-regulation appeared in 9% of posts, career, 6% of posts, and socializing, 4% of posts.

**Discussion:**

The findings challenge the traditional deficit perspective by demonstrating that focused interests in autistic adults are characterized by knowledge seeking, a construct similar to systemizing, but more explicitly on a continuum with academic interests. These interests are not restricted but wide-ranging, resembling the explorations of polymaths. The established view, that intense interests reduce anxiety by promoting predictability, originated in historical attention to autistic individuals with atypical intellectual ability. Intellectual disability obscured how repetitive behaviors such as object manipulation are ‘experiments to see’. I propose that knowledge-seeking is the primary function of systemizing and intense interests, thus unifying focused interests across multiple levels of intellectual ability of the autism spectrum.

## 1 Introduction

You narrowly missed my original title: *Who has circumscribed and restricted interests? Career-minded academics.* After that title, I planned to open with: Apologies for the clickbait title, but now that I have your attention, let me acknowledge that it is autistic individuals who are most tightly associated with the terms circumscribed and restricted interests (e.g., 1–4).

Prudence prevailed in title choice. In recent years, the understanding of autistic interests has shifted to incorporate strength-based perspectives (5–7). Theorists have noted that it makes more sense to characterize these focused interests as intense and idiosyncratic, rather than specifically restricted (8, 9). In this article, I go one step further. While the interests of autistic adults are often highly specific, they are also wide-ranging and intellectually oriented. That is, the opposite of restricted and circumscribed.

My point is to enlarge our understanding of the interests of autistic adults with normal intelligence given that this group is approximately 60% of the autistic population (10). Hereafter, I will refer to this group as autistic adults. I seek to document that while autistic adults’ interests are often idiosyncratic, specific, intense (and sometimes interfering), they also have an overlooked attribute: they are intellectual and focused on knowledge acquisition. This perspective builds on the *interest-driven mind* discussed *in* Murray et al.’s (11) theory of monotropism. I maintain that knowledge-seeking and ‘experiments to see’ also motivate repetitive object manipulation and unusual interests in autistic youth and autistic adults with intellectual disability. Knowledge-seeking be conceptualized as a core autism characteristic, but establishing this requires separate analyses (e.g., 12, 13).

But why did I bring in the ‘restricted interests’ of career-minded academics? The interests of autistic adults resemble those of scientists and academics by being highly specialized and intellectual, yet are frequently also wide-ranging by including many different disciplines. As I document in this article, the same autistic adult frequently pursues topics as diverse as academic-style readings about marine biology and Victorian-era architecture. When non-academic topics are the focus of interest, autistic adults will often pursue these in a manner that resembles the knowledge-seeking of professional researchers.

### 1.1 The traditional view of autistic interests: circumscribed, narrow, restricted

Autistic interests have traditionally been referred to as circumscribed, narrow, and restricted (8). Examples from individuals with intellectual disability and/or high support needs are train schedules, WWII bi-planes, ceiling fans, and washing machines (14). A 12-year-old autistic boy would speak nonstop about airplane windows; a 5-year-old was obsessed with storm drains. Much has been written about how intense interests disrupt family life, interfere with socialization, and impair classroom learning (e.g., 15–18).

Theorists have long been flummoxed to explain the purpose of intense and focused interests (17). The most frequent explanation is self-regulation; emphasizing sameness reduces prediction error (19). Variations of this theme include reducing stress by performing a well-known activity (2, 20); avoiding demands such as social activities (16, 21); seeking a calming, regulating activity (17), and minimizing boredom (9).

Less commonly mentioned is that special interests afford systemizing, a strength of many autistic adults (5, 22, 23). Summarizing the conclusions of authors such as Winter-Messiers et al. (14) and Klin et al. (1)Lung et al. al (, 3, p. 117) wrote, “Passionate pursuit of these interests enabled autistic persons to understand and interact with their environment, fostered autonomy, and provided a sense of personal validation.” Passionate pursuit of interests is a repeated emphasis in Wenn Lawson’s (24) book, *The passionate mind: How people with autism learn* (see also 25). Lawson elaborates the strength-based view of autistic focused interests called monotropism (11). Monotropism proposes that the core characteristic of autism is the *interest-driven mind.*

Dachez and Ndobo (9) also emphasized the sense-making function of interests, as in attempts to understand the world. They concluded from interviewing autistic adults that:

…their special interests enable them to have a better understanding of human beings and social relationships. The human being thus becomes a subject of study, through the analysis of psychology, for example. It is a strategy oriented towards problem-solving, as social difficulties represent the core of autistic symptomatology and are the cause of the situations of exclusion experienced. By becoming interested in the human sciences, they try to compensate intellectually for what they cannot achieve innately. By understanding better how humans function and the underlying mechanisms of interactions, they try to find solutions to adapting and integrating (9, p. 89).

I will revisit this point later as it accords well with the findings in the current study.

Cascio et al. (15) summarized diverse studies suggesting that autistic individuals find their focused interests to be positive, engaging, and enjoyable. But authors have admitted to being perplexed about many aspects of focused interests, noting that focused interests “…remain under-researched and poorly understood” (4, p. 1296). Harrop et al. (26, p. 1797) wrote: “…as a field we do not understand what causes RRBs [Restricted and Repetitive Behaviors].” Cascio et al. (15, p. 163) noted: “Restricted interests … can manifest as common, age-appropriate hobbies (e.g., a particular video game in adolescence)… but there is little empirical evidence for whether or how that may differ from typical hobbies and interests.”

I previously investigated how autistic interests differ from neurotypical hobbies and interests. Autistic-identified adults, compared to neurotypical (NT) adults, reported more specific interests, more interests in systemizing domains, and a greater number of interests overall (23). Other researchers have since confirmed these findings with interview data (9), as we also did with a survey (22). Chloe Jordan and I proposed that the degree to which interests are guided by the exigencies of socializing vs. systemizing is what distinguishes autistic interests from NT hobbies. In our 2014 survey, the most frequent NT interests were music, sports, food, and entertainment. Stating one’s interests at a broad level facilitates inclusion and connection with others. We concluded that NT interests are low in specificity and highly frequent in the environment (e.g., music, sports and entertainment) because these interests maximize social opportunities (22). The big-three of NT interests (music, sports and entertainment) only secondarily meet the autonomy and competence needs described by social determination theory (27). Large individual differences exist in these pursuits across neurotypes. Many people strive to make one or more of music, sports or entertainment satisfy multiple needs, including career and rewards from daily involvement (28–30).

But whether interests are broad or restrictive reflects the same two factors in both autistic and NT adults. These are individual differences in wanting to be selective about using interests to signal and establish social connections, and one’s own cognitive comfort zone. The cognitive comfort zone is the grain size of information, which is most comfortable to process, ranging from narrow and algorithmic (e.g., math, computer programming, science) to fuzzy, undefined and cross-cutting domains (e.g., *fiction, philosophy, political affairs*). This notion overlaps with the concept of weak-to-broad central coherence as a cognitive style (31), not a cognitive deficit. For autistic individuals with intellectual disability, examining overhead light streaming between one’s fingers allows the amount of light to be dynamically varied, constituting an experiment on the physical world (12).

### 1.2 Current study and gap in the literature

To hear directly from autistic individuals themselves, I turned to forum analysis. Forum analysis complements surveys and interviews with the advantage of bypassing leading questions and interviewers’ agendas (see summary of advantages and disadvantages in Table 4 in 32). Key questions representing gaps in the literature are:

1. Are special interests primarily restricted to domains that afford systemizing, as assumed by prior research, and also as in the stereotype of autistic adults (stereotypes of computer systems and mechanical objects, discussed by diverse authors such as 14, 33, 34)?
2. What evidence was present for interests being restricted and idiosyncratic, as assumed by autism diagnostic guidelines and intense, as mentioned in writing about monotropism and in research with autistic youth and adults (8, 9).
3. What functions are being served by the interests, as described by posters? I investigated whether posters discussed how their interests serve functions identified in the prior literature (e.g., 3). These were:

- Knowledge seeking (also: systemizing; 22),
- Mastery (includes autonomy, sense-making and career-interests; 3; Dachez & Ndobo; 1)
- Self-regulation. This includes anxiety reduction and escaping demands (e.g., 1, 2, 16, 20), but also promoting predictability, 19)
- Socializing (interests facilitate social connections, see Grove et al., 14, 35)

Consistent with the last bullet point above, my undergraduate research interns and I performed content analysis to determine whether posters mentioned interests serving socialization goals. This of interest because it is the primary purpose of hobbies and interests for neurotypicals (22), and but autistics’ intense interests have long been considered socially isolating (e.g., 8).

A fourth question was:

4. What attitudes or other experiences do posters mention when discussing their special interests?

## 2 Methods

### 2.1 Selecting platforms, forums, and posts

To capture the unmediated perspectives of autistic adults, this study used an observational netnographic approach (36, 37). Netnography adapts traditional ethnographic research principles to investigate computer-mediated communication within digital cultures. My team of undergraduate interns, naïve as to specific research questions, conducted a search for discussion threads on Reddit, Quora, Wrong Planet, and Twitter. These platforms primarily contain written postings in a question-answer format. All posts on these platforms are publicly accessible. Methodological details were informed by my lab’s prior research on forum analysis (23, 31, 32, 38, 40).We selected a target of 260 posts as a number large enough to extract generalizations but small enough to be tractable, following prior analyses by our team. Team members searched within the platforms using search terms such as *special interests* and *hyperfixation*. To find additional posts, our team also utilized the ‘related posts’ feature available on Quora and Reddit. To select posts systematically, all posts available in an identified discussion thread were pasted into an Excel spreadsheet, as long as the title of the opening thread referred to interests, or interests were salient within the responses to an opening thread, as in these examples.

I was obsessed with reading, cats, whales and different coloured hair.

Wanted to show another one of my texture paintings. Its my favorite thing to paint right now because its so calming.

This method resulted in 260 posts being extracted from 8 forums. **Table 1** lists platforms, forums and the specific questions that headed the chosen discussion threads. The top half of **Table 1** lists 8 opening questions that explicitly mention special interests, along with the number of posts that responded to that question. The majority of posts in our corpus (249 out of 260) responded to broad questions such as *What are your special interests?* The bottom half of the forum lists a less numerous set of discussion threads (N = 21) that did not open with a question about special interests, but posters discussed their interests as part of responding to the opening question. This was particularly common for threads about jobs and careers, as respondents advised getting a job compatible with one’s special interests. We included these posts in the corpus to be as inclusive as possible.

**Table 1:** Sources for the 260 posts.

Platform	Forum	Question that opened the discussion thread	
Forums where there the opening question concerned interests			N
Reddit	Aspergirls	What are your special interests at the moment?	55
		I’m curious -- what are your current special interests?	48
		What is the difference between ASD special interests and NT hobbies?	43
	Autism in Women	What are some of your hyper fixations/special interests?	48
		What are your special interests?	25
	Autism	Anyone else really embarrassed about their special interest?	2
	Ask Autism	My LDR partner’s special interest is their work	2
Wrong Planet	Adolescent Autism	What are some of your special interests or hypersensitivities?	16
Forums where interests were mentioned or discussed in body of the post			
Reddit	Aspergirls	Success stories of women on the spectrum	5
		Autistic career success stories	4
		Job fields for women on the spectrum	2
	Aspergers	Does anyone have serious trouble finding a job?	2
		Any other women with high functioning Aspergers?	2
		I like being autistic	1
	Ask Autism	How important is early intervention?	2
Quora	Autism and Girls	Could a person have no symptoms until age 11?	2
Twitter	Autistic Girls Network	…among autistic girls [books] are a way to escape the world…	1
Total Forums 4	Total Forums 8	Total Discussion Threads 17 Total Discussion Posts	260

The mean length of posts was 64 words (median: 40 words, range 1-350; except for 2 posts had much longer lengths of 708 and 742 words). The entire corpus was 17054 words; we omitted the final 54 words of the longest post to obtain the convenience of a corpus with 17000 words. Binning the corpus into quartiles revealed that 25% of posts were between 1 and 18 words in length, the next quartile contained posts between 20 and 39 words, with the third and fourth quartile having posts containing 40 to 75 words and 76 to 350 words (excepting the two longest posts), respectively.

### 2.2 Content analysis

The posts’ contents were analyzed using content analysis (41). The author and research interns read through posts pasted into an Excel sheet, highlighting statements that were the main ideas of the post. From these, our team discerned broad topics and specific topics within these (**Table 2**).

**Table 2:** Taxonomy of topics and percentages of posts with these topics; Some topics have subtopics.

Academic topics (4)	Total 56%	Frequently mentioned topics; Misc. quotes
Psychology, Neurology, Sociology, Autism	29%	Neuropsychology, Sociology, Mental Health, Autism, Personality Disorders, Human Sexuality, Cultural Psychology, Cults, Narcissism
Science, Medicine	20%	Geology, Physics, Biology, Microbiology, Chemistry, Diseases, Health Care
History, Culture, Literature	18%	General History, Ancient Civilizations, History of a Specific Region (Africa, Scotland, China), General Literature, World Religions, Ancestry
Linguistics, Languages	12%	Linguistics, Learning Languages, American Sign Language, Speech Pathology, Writing, Reading
Entertainment (4)	Total 46%	
TV, Movies, Videos, Music	29%	Specific Movie and TV Series (Star Wars, Harry Potter), Anime, Listening to Music, Playing Instruments
Books, Literature	14%	Books in General, Specific Series, Romance Novels
True Crime	9%	True Crime Documentaries and Podcasts, Famous Murder Cases, Wrongful Convictions
People and Celebrities	5%	Specific Actors, Artists, Musicians, Content Creators
Leisure activities (3)	Total 40%	
Artistic Interests	21%	Drawing, Painting, Digital Design, Photography, Quilting, Knitting, Stitching
Video games, Technology	21%	Software, IT: Video Games and Game Design
Games and Toys	7%	Toy Collecting, Legos, Boardgames, Squish Toys
Animals (3)	Total 29%	
Cats	18%	*“Seeing a lot of cats here! Obsessed with my kitty.”*
Dogs	6%	*“Dogs; purebred dogs and their traits and characteristics.”*
Other	22%	Aquariums, Animal Behavior, Wildlife; Other Pets
Lifestyle (3)	Total 28%	
Nature, Exercise	15%	Gardening, Plants, Sustainability, Environmentalism, Running, Hiking, Cycling, Sports, Being Outdoors
Food	8%	Cooking, Baking, Specific Foods, Specific Diets
Beauty, Fashion	10%	Clothing styles; Skincare, Makeup, Nail Polish
Misc. Comments	Total 19%	Advice; Female Presentation; Niche Interests; General comments on special interests and their intensity; Insights about how autistic SIs differ from NT hobbies

Most posts mentioned several interests; totals sum to more than 100%.

To address question (1), team members categorized interests and grouped them into larger categories such as music or health science. New categories were created when recurring interests were identified during reading and discussion of the posts. A taxonomy of types of special interests evolved as the team discussed topics during lab meetings. This taxonomy appears in **Table 2**.

To address question (2), we coded posts for the pre-identified functions listed in question (2) (**Table 3**). Team members also took note of any additional recurring themes in an exploratory manner.

**Table 3:** Themes and sub-themes identified in the corpus of 260 posts, organized by frequency.

	Function or purpose of special interests	%
*Knowledge seeking*	Allows systemizing (collecting, annotating, organizing)	28
	Researching society and human behavior (improves relationships, understanding of groups)	26
	Intellectual approach to conventional content	25
	Wide-ranging intellectual appetite	20
	Learning for its own sake	12
*Mastery*	Obtain expertise	16
	Enables job opportunities; workplace success	6
*Self-regulation*	When bored, special interests stimulate	7
	When stressed, special interests calm	2
*Socializing*	Find friends who share one’s special interest	3
	Strengthen friendship via sharing interest	1
	Additional frequently mentioned themes	
*How Interests change*	Current interests vs. prior interests	30
	Change in interests over one’s lifetime	15
*Feelings about one’s interests*	Intensity and enthusiasm	33
	Humor, irony, pathos about post content; used *lol* or *ha ha*	12
	Having an odd or unacceptable interest	9
	Large amount of time spent on interest	8
	Interest interfered with daily life or caused distress	5
	How interests connect with each other	4
	Wanting to be done with an interest	3

Most posts were coded as having several themes; totals sum to more than 100%.

After the strong focus on learning was identified during thematic analysis, I sought to quantify how strongly special interests serve knowledge acquisition by reading through posts to identify words connected to knowledge, such as *learn* and *study*. Morphological variants of words were included in counts when meaning was congruent, as in *I want to study … I studied … I am now studying …* Words were also identified corresponding to the intensity of interests. A similar tallying procedure was used for words relevant to the functions of socializing, job/career, and stress regulation. Because common words are highly polysemous, the word count procedure took into account the meaning of the word in its context. As an example, the keyword *social* listed in **Table 4** was counted as facilitating socializing in examples such as these: *researching how to refine my social skills to make friends more easily* (although note this is an interest in social skills as an intellectual topic, not interests as venues for socializing). I excluded uses of *social*, such as *social justice, social psychology*, and *social science*. Similarly, occurrence counts excluded uses of the word *learn* that were not related to interests, as in this example: *The only reason I can pass as neurotypical is because I have spent many difficult years being forced to learn how to act ‘normal.’*

**Table 4:** Word counts supporting functions of special interests.

Knowledge seeking		Intensity		Socializing		Self-regulation		Career	
	%		%		%		%		%
learn[s,ing,ed]*	13.3	love	17.6	friend	10.3	stress	2.4	job	9.7
read	11.5	years	12.7	social	3.6	calm	1.8	career	3.0
science	6.1	obsess	7.9	share	3.0	escape	1.8		
research	4.8	fun	7.8	bond	0.6	relax	1.2		
Stud[y,ied,ying]*	4.2	enjoy	4.8						
brain	3.6	super	3.0						
write	1.8	fascinate	3.0						
figure	1.8	rabbit-hole	1.8						
facts	1.8	focused	1.8						
knowledge	1.8	deep	1.2						
analyze	1.2	binge	1.2						
		hooked	0.6						
*% of posts with one or more terms*	52%		45%		6%		3.8		3%

*Two of the words were frequently used in different grammatical roles, and thus the root morpheme (or lemma) was used in these tallies.

## 3 Results

### 3.1 Taxonomy of special interest topics

Special interests were not restricted to domains that afford systemizing, allowing us to qualify some prior research and stereotypes about interests (e.g., 33). Nonetheless, systemizing was frequent (see section 3.2.3).

#### 3.1.1 Academic topics

Academic topics dominated posters’ special interests, appearing in approximately half of all posts. These were categorized into 4 topics listed in the top panel of **Table 2**. The most frequent of these, the broad category of psychology-related topics (Psychology, Neurology, Sociology, Autism) was mentioned in more than 1/4 of all posts.

Why psychology is so appealing is identified in next two posts, both appearing in forums for autistic women:

…attempting to dissect socio political themes and views in various forms of media and dissect the psychology behind narcissists.

I like researching about autism and psychology in general, and neuroscience. I also get very into reality shows on YouTube because I think I just like studying people’s lives without really engaging with them.

My team inferred after reading many posts that psychology is of intense interest because it allows autistic adults to use their systemizing abilities to decode the social world, as also noted by Dachez and Ndobo (9). The next post illustrates this in an indirect manner. The topic of the US Space Race appears to fit the stereotype of autistics’ interests in science, but the female poster flips this script and notes that her interest is in the social world.

My special interests tend to be people-based rather than thing-based. For instance, take my fascination with the Space Race/Apollo lunar missions. I’m not very interested in how the Saturn V rocket worked … I’m mostly interested in the lives of the astronauts themselves and their families.

Although the poster does not say this directly, reading about astronauts’ families provides social data from which one can extract generalizations and make inferences about the social world.

Topics related to science were the second most common academic topic, mentioned in one-fifth of posts. Subtopics covered myriad scientific domains, with posters often appearing to revel in the intensity of their interests, as in this example:

I can spend hours reading about cell biology, endlessly fascinated. My latest hyper fixation, however, was about a protein called focal adhesion kinase and I spent hours learning everything I could about it and figuring out how it would look like in a cell visually, so I drew lots of diagrams.

What is noteworthy here is a level of interest which mirrors how scientists (or science students) approach the topic.

History, culture and literature topics were the third most common academic category (18% of posts). The final broad category of languages included learning languages as well as sciences related to language (such as linguistics and speech pathology). These topics also appeared to allow systemizing of the social world.

References to language often gave enthusiastic and specific details of the language of interest, and mentioned being self-taught, as in:*…languages (learning Spanish/French in school, learning Japanese/Russian/Norwegian on my own)!*

One poster described: *I find grammar and structure sooo fascinating*!! Enthusiasm about language learning is at odds with the older view of language as a deficit in autism, but has been observed in forum analyses specific to second and foreign language (39, 40).

The special interests of reading and writing are included as a sub-topic in this category but note that only 4 posters listed reading as their special interest writing, and only 3 posters listed writing (1% of all posters).

#### 3.1.2 Entertainment

Based on frequencies of mention, it made sense to organize non-academic topics into the four broad categories of Entertainment, Animals, Lifestyle, and Leisure Activities. The largest category, Entertainment, featured TV, movies, videos, and music, similar to NT interests. Science fiction and fantasy shows were frequently mentioned. Posters frequently discussed studying entertainment media as if it were an academic topic, as in *I asked for a book with all the episodes and summaries and quotes…*.

#### 3.1.3 Leisure activities

Artistic interests were mentioned in approximately 15% of posts, a frequency similar to the Science, Medicine category. Our team agreed that these posts often resembled our intuitions of how NTs would describe artistic hobbies. For example, one poster wrote, *I am a lifelong artist, I pursue it thru drawing, writing, every kind of art craft.*

In the Games and Toys subtopic, highly specific collecting was mentioned, as in these examples:

collecting bat figurines; collecting/caring succulents; collecting gastroanthropology

The broad category of animals was mentioned in 29% of posts. Comments were diverse, but comments appeared similar to how NTs regard their pets, as judged by our team. Nonetheless, many posts discussed highly specific interests, such as this post about purebred dogs:

I can recognize just about any of the AKC registered breeds and know their breed standard and common characteristics. Many I can identify just by a partial view of their body such as the tail.

Interests in animals was often broad and diverse, as in this post.

animals and nature in general! as a kid it was mostly wild animals and bugs/critters. i had millions of bug boxes throughout the years to put frogs and other bugs in. i loved snakes for a while, then big cats, and most recently researching how to care for various pets and invertebrates … i met a pig today for the first time and i think they’re going to be my new focus because aaaaaa!!!!!

Greater interest in cats than dogs among autistics has recently been investigated and explained as cats allowing a more reserved and less demanding or less boisterous interaction style (42).

#### 3.1.4 Lifestyle

Within the subtopic of gardening (categorized under Nature), examples abounded of specific or unusual plants (such as succulents or fungi). In some cases, plants had become projects:

I currently have an “office” which has just become a greenhouse where I am propagating a bunch of plants, not including all my gardening stuff I have planned for next year.

Posts in the categories of food also often had a ‘project’ focus, where the food was wrapped up in larger goals.

Currently it can be summarized as trying to live as sustainable/self-sufficient as I can. Fermented foods (from theoretical to the practical side of it) I make my own bread, sauerkraut, yoghurt and brew my kombucha. I also started my own vegetable garden. Every time I grow/make something instead of buying it I feel like I’m hacking “the system” and I find it fascinating.

Food topics also often had an academic and science angle:

I love … all sorts of cooking/food related facts, especially pertaining to sociological/historical aspects.

### 3.2 Interests are not restricted; sometimes idiosyncratic, but overall, characterized by knowledge seeking

Idiosyncratic interests were frequent. Yet the interests were the opposite of restricted. Many posters described a large number of interests. Posters’ interests were not just intellectually oriented, but wide-ranging in how they spanned the taxonomy of possible human interests, with the same person frequently having interests from diverse domains.

At the moment I’m really focused on recipes from the depression era. I’m also focused on people leaving California due to the economy, and gentrification. Plus just recently I’ve been obsessed with the LAUSD [Los Angeles Unified School District] strike.

The above post is an example of wide-ranging intellectual orientation, since interests are noted that span 4 topics in the History and Culture category. History (Depression era) and Culture (leaving California, gentrification, LAUSD strike).

As noted above, intellectual interests predominated. However, beyond the presence of academic topics was a drive to learn new information that I term *knowledge seeking*.

Here, I describe four themes that capture distinct aspects of the omnipresence of knowledge seeking in the corpus.

#### 3.2.1 Intellectual approach, even when topics were not conventionally intellectual

It was striking when an intellectual approach to topics such as reading, movies, cooking, gardening, and pets. Posters often mentioned studying these or investing systematically in the knowledge systems behind the topic, as in these three posts about *fictional worlds, cooking*, and *beauty.*

I really like analyzing the worlds/societies of fictional media. I always have questions about it, or want to debate all about it with others, especially from a sociological/anthropological perspective.

Cooking/food related facts, especially pertaining to sociological/historical aspects.

Many of my revolving special interests have to do with the beauty industry. Right now I’m enjoying the history of the cosmetics industry in America and finding the parallels between our attitudes about beauty in the past vs. today.

#### 3.2.2 Wide-ranging intellectual appetite

When reading posts, our team was surprised by the number of interests and also their diversity. Posts described interests that spanned disparate categories. Below are responses to the question, *What are some of your hyperfixations/special interests?* These are the first five responses to the opening question in this thread; I did not purposefully select the most striking posts.

They come and go, but in the past few years: theology, greyhounds, several works of fiction, Irish history, Inquisition history, theoretical physics re: time, genealogy.

-Health science/diseases, disorders, etc. -Little house on the prairie -Cooking -History -Shows based on a specific time period past 1960s

Guinea pigs (I have 3) and nail polish (I have over 100)

Mine at the moment are astrology, numerology, skincare, and geography/biomes! I also really like flags, and memorizing them all, if you show me a flag I can most likely tell you where it’s from!!

I also love knitting and crocheting and all sorts of cooking/food related facts, especially pertaining to sociological/historical aspects.

I categorized a post as indicating *wide-ranging intellectual appetite* if it contained at least 4 categories from different domains. A domain refers to different academic domains or different major categories in **Table 2**. For example, natural sciences, social sciences, art and fashion are different academic disciplines. Interests in applied and non-academic arenas are also counted as a different domain. For example, this poster mentions more than four distinct domains.

Natural sciences (yes, all of them, but especially geomorphology, meteorology and biogeography), medical science (particularly epidemiology, lately), cognitive science (especially developmental psychology), social sciences (especially relationships), economics (especially behavioral economics), art, architecture and design (everything from industrial design to automotive design to fashion to typography).

The next three quotes are additional examples of having interests in four distinct domains.

Animal Crossing: Pocket Camp - I just found the subreddit for it. Social attitudes regarding disability, both visible and invisible, and the consequences, positive and negative, of accommodation. Food and agriculture law in the United States. And birds, always birds. I had a dinosaur phase recently, and that may be making a resurgence soon):. [video games, social attitudes, agricultural law, and birds]

I love philosophy and art, with sociological aspects. I’m especially interested in visual culture and films, the topics are so vast. I also make stop frame animation videos using random objects (because I become fixated with specific objects). [philosophy, art, sociology, film]

Right now mine are falconry (I’ve had this interest on and off for seven years), the Norwegian language (I’ve been into Germanic languages for a few years and bounced from German to Swedish to Icelandic and finally I’m starting a class in Norwegian), linguistics in general plus I’m trying to get into conlanging, and I’m starting to develop an interest in 1930s fashion. I also have an unhealthy interest in true crime. [birds, languages, fashion, true crime]

#### 3.2.3 Systemizing

Systemizing was prominent in many posts. The research literature on autistic interests highlights systemizing with respect to interests in math, science, machines, and computer programming (e.g., 5, 33). Posters in our corpus described these topics but also applied systematizing to the social sciences, as in this post about military history.

I’ve been studying historical military systems, mainly how different armies and empires, as well as just cultures and war bands handled their logistics and general troop movements, a lot of different armies from different time periods had novel answers to huge ranges of conditions, it has given an interesting perspective on how this aspect of their lives was formed and led.

Systemizing was also applied to exploring the social brain, as shown in this post about true crime.

I also like true crime; but ones where it digs into the why behind everything…. how the FBI created the behavioral crime unit … to understand how/why serial killers function. I like the show because it really delves into the psychology of how their brain functions.

This is consistent with the observation of Dachez and Ndobo (9), that interests in the true crime genre allow autistics to problem-solve about complex and non-socially acceptable human motivation.

#### 3.2.4 Learning for its own sake; craving information

Posters described an enjoyment of learning and indeed a drive to take in information, as in these examples.

Learning (and usually immediately forgetting) stuff about plants & fungi.

Seems “learning” is my deepest special interest, haha I will watch literally any nonsense on YouTube to fill my brain.

My special interests go through phases too. My current is learning about autism, executive dysfunction, and things similar. Can’t say I’m an expert, but I enjoy learning about it! Before that, I’d go through artistic phases (music, writing, drawing, etc.) and other educational focuses.

### 3.3 What functions are served by focused interests

The prior section presented evidence that a primary function is knowledge seeking. Here, I review evidence for other functions noted in the literature.

#### 3.3.1 Mastery and career

The functions of gaining expertise and its application to career (coded as mastery) were frequently mentioned in posts, **Table 3**. Posters frequently mentioned the rewards of leveraging interests for a career.

I got a job in a field that lines up with one of my special interests … easier for me to blend in as I pretty much get paid to talk about and work on my special interest with people who are also heavily interested in it. Helps me keep from getting autistic burnout and because it’s something I’m so passionate about, it helps me excel compared to others…. My advice for Aspies is to get a job somewhat related to your special interest.

I found two things I’m really attached to and can do successfully … I’m sticking with them. I went through a lot of jobs to get here, some even lasting a few years … Now I work with kids at a library and it’s pretty stress free and I’m good at it. But you’re not weird if you’re having trouble … that took me until I was 37.

These next two posts noted that every job choices has its downsides, but turning interests into a career is worth managing those challenges.

I’m a photographer for a major retail company and get to travel all over the country! Although the travel is stressful I just find my ways to cope with it … Photography is extremely technical and that’s why I love it, I turned my passion and special interest into my career!

Long-term, I need to work in a field which is of interest to me to be happy, and speech/language is my biggest special interest. Yes, there’s a lot of communication (duh), but sessions are planned out and essentially scripted which makes it way easier and far less exhausting for me.

#### 3.3.2 Self-regulation

Following the literature, I identified posts where special interests were described as being calm, relaxing, and used to escape or reduce stress. For example:

Books are a common special interest among autistic girls. They are a way to escape the world, when life is too overwhelming. Thank you to all of the well-loved stories which made life a little easier growing up.

#### 3.3.3 Socializing

Posts rarely mentioned that special interests could be a vehicle for connecting with others (see **Table 3**). The only three examples of this are the following.

Make friends with those who share your special interest

Try making friends based on your special interests, and then meet the friends of those friends!

I would suggest seeking out other people who share your weird interests, and with whom you can enjoy talking about them. While shared interests, alone, are not a guaranteed basis for friendship, they can certainly be an excellent first step towards friendship.

This next poster despaired that her interests were so specialized that no one would share them (which relates to the idiosyncrasy of interests).

When I want to make a fan page online for my special interests, I always feel guilty about it … It’s a very, VERY lonely feeling. All I want is someone with the exact same interests as me, someone I can talk about my interests with all day and feel comfortable doing it…

One poster mentioned that an interest increased social bonds.

Ancestry [23&Me] for myself and a few family members for Christmas … I even got my dad (also has ASD) in on it … A fun way to bond.

One poster mentioned wanting to learn about social skills, while also implying this hadn’t achieved its intended goal: *researching now to refine my social skills to make friends more easily (while also simultaneously disliking and avoiding almost all people)*.

Two posts mentioned that interest in animals took the place of human friendships.

Also I have 3 rats that I’m obsessed with everything about them. One time my partner joked that I have rats instead of friends and it was intended to be a lighthearted joke and instead of getting offended I had an incredible realization that he is right and i completely embrace it now! Haha!

I just really like cats, my cats, everyone’s cats, cat memes, etc. I have to stop and try to befriend every cat I see outside. I know more of my neighbor’s cats than me neighbors.

One poster referred to the strategy *Develop interests that are popular with others* but noted that it did not succeed for her.

I was assessed in kinder but my parents were told I was just shy and needed to find an activity to make friends. I did 12 years of ballet but came no closer to making any friends.

It is worth noting that this poster feels that in childhood her autism was mistaken as shyness, a frequent topic in online discussion boards about why women are underdiagnosed (e.g., 12).

This same poster goes on to share that joining popular activities didn’t help her gain friends.

I was obsessed with reading, cats, whales and different coloured hair. I got involved in drama and played percussion in high school but was bullied and didn’t make friends.

This next post is one of the few noting that an activity with a friend led to a special interest.

I also co-write a story with a friend of mine which has become a special interest of mine.

#### 3.3.4 Quantitative analysis of words relevant to functions served by interests

The first four columns in **Table 4** list the percentage of posts containing at least one word related to the four candidate functions of intense interests. Knowledge-seeking was highly evident in the corpus, with 52% of posts containing one or more of these terms. In contrast, words relevant to socializing occurred in 6.4% of posts. Words related to self-regulation and career occurred in only 3.8% and 3.0% of posts, respectively. Also shown in **Table 4** (right-hand column) are percentages of posts containing words suggesting emotional intensity. Almost half (45.4%) of posts contained one or more of these words.

### 3.4 Additional frequently mentioned themes

#### 3.4.1 Having an odd or unacceptable interest

The bottom panel of **Table 3** lists themes extracted during analysis. These varied themes were categorized as *Feelings about one’s interest.* An example is *Having an odd or unacceptable interest.* Posters remarked that their interests were unusual or niche interests, such as *the kind of interests that make NTs look at you funny.* Examples in this category include:

Anyone else feel really embarrassed about their special interests- like never wanting to share them with others? Whenever someone asks me what my special interests are, I always answer dishonestly.

#### 3.4.2 How intense and unusual interests are used to speculate about an autism diagnosis

Intense interests are classified as part of the broad category of ‘restrictive and repetitive behaviors’ which constitute one of the defining characteristics of an autism diagnosis. It is thus noteworthy when people observed that the qualities of their interests suggests they may have autism (as in first quote below) or may not have autism (second quote).

This poster noticed that the intensity of interest they experienced eclipsed what they observed in others, which made them wonder if they were autistic.

So, gaming is my special interest and my involvement with it was a big reason I started to question whether I might be Autistic or not. I don’t have friends so I’ve tried to bond with people over gaming but it’s never quite right for lack of a better word. I’m always too extreme with it apparently … So I’ve met some NT people who … are as familiar with the indie scene as I am but at the end of the day we never connect because I’m always too into gaming, even for the more extreme hobbyists…

Here an autistic woman reflects that her interests in communication caused her mother to dismiss autism as a possible diagnosis.

One of my special interests is problem solving and interpersonal communication type things. I don’t remember the exact name of it but it’s in the book “Siblings Without Rivalry.” I’ve read it like 10 times. I’m better at dealing with my little siblings than my mom is, because I follow the exact wording and steps the book tells me to … My mom says she doesn’t think I’m autistic because I focus so much about interpersonal relations and stuff and if you’re autistic you focus more on mechanical stuff and numbers.

## 4 Discussion

I first review how the findings bear on the conceptualization of autistic interests in research, beginning with the function of intense interests followed by why mainstream autism research has mischaracterized interests. I then delve into implications of these findings for diagnosis, clinical support, self-understanding, and career considerations. I structure the discussion of career considerations in terms of opportunities and barriers, using academia as a case study for exploring these topics. **Table 5** summarizes some of the unique take-aways of this project.

**Table 5:** Implications.

Short form	Key take-away
Intellectual curiosity	Autistic interests are best understood as a systemizing process embedded in intellectual curiosity.
Similarity across intellectual ability levels	Even RRBs that are typically pathologized (such as a fascination with observing light through wiggling fingers), can be understood as an intellectual exploration of the world.
Explaining to peers vs. researchers	How autistic individuals explain their interests when interacting with peers online can differ from how they respond in qualitative interviews with researchers.
Intrinsic vs. extrinsic interests	Inherent interest vs. social acceptance influence the content and exploration approach of both autistic and neurotypical interests, but intrinsic interest may play a greater role for autistic people.
Motivation for interests can illuminate diagnosis	The underlying motivation for engaging in interests, as well as the integration of an interest into other aspects of a person’s life, should be considered in diagnostic practices.

RRBs, restrictive and repetitive behaviors.

### 4.1 Conceptualization of autistic interests in research

#### 4.1.1 Wide-ranging and intellectually oriented

The interests identified in this analysis of online discussion forums were frequently intellectual in nature, resembling the specialization of scientists and scholars. These findings are consistent with a growing trend to move away from the classic and older characterization of interests as restricted, circumscribed, and idiosyncratic. This point was forcefully made by Grove et al. (21) but has also been mentioned by Anthony et al. (8) Dachez and Ndobo (9), and Nowell et al. (16). For example, a sample of children (mean age 9) studied by Nowell et al. (16) reported interests in astronomy, geology, physics, history and psychology. In a comparison of NT and autistic interests (age 7–24 years), Anthony et al. (8) identified academic topics that were similar in intensity between NT and autistic respondents. These included physics, psychology, astronomy, geography, and reading.

More importantly, when interests were not stereotypical domains that afford systemizing (meaning, mechanical and other natural systems), posters nonetheless frequently took an in-depth, scholarly approach. For example: cooking/food-related facts, especially sociological/historical aspects. This is the stance of social scientists, where everyday topics are studied from the vantage point of a scholarly discipline. Consider also this example from the cooking/food category: *Bread making and the science behind it. It infuriates people. LOL*. The poster here implies that bread-making is a socially acceptable interest, but being interested in the science behind it is less typical.

Another striking characteristic that has been less mentioned in the special interests literature is having diverse interests, which I labeled *wide-ranging intellectual appetite*. These resembled the knowledge seeking of a polymath, a point discussed in Fitzgerald and O’Brien’s (43) book on intellectualism in autistic adults.

#### 4.1.2 The primary function of intense interests: learning and domain exploration

The posts analyzed here provide compelling testimony that the primary function of interests is learning, exploring, and discovery, as shown in frequency tallies in **Tables 2**-**4**. Posts rarely discussed self-regulation, escape, and socializing. Yet, outside of theorists who emphasize systemizing (e.g., 33), self-regulation and reducing uncertainty are presumed to be the dominant purpose of intense interests (1, 2, 4, 16, 19, 20).

### 4.2 Why has mainstream autism research mischaracterized intense interests

#### 4.2.1 The legacy of the deficit perspective

I drew on the forum responses to conclude that autism research has mischaracterized intense interests as self-regulation, demand avoidance, or representing a systemizing compulsion. Why has mainstream autism research avoided characterizing intense interests as centered on knowledge seeking? The main reason is the deficit perspective (44). The field of autism research originally focused on children and adults with high support needs, which often included intellectual disability. Such individuals also have deficits in social understanding, such as could occur with decreased endogenous rewards originating from social stimuli (45–48). The deficit perspective is a reasonable one when working with children and adults with reduced cognitive and social processing ability. Historically, the concept of knowledge-seeking may have been viewed as incompatible with lower cognitive ability, making it hard to perceive knowledge-seeking even when one is confronted with intellectually able autistic adults, as some theorists have long recognized (e.g., 7, 49, 50).

#### 4.2.2 Extending knowledge-seeking to autistic individuals with intellectual disability

But assume with me for a moment that knowledge seeking (meaning, moving beyond just systemizing) is one of two core characteristics of the autism phenotype. In the face of constrained (and even severely constrained) cognitive ability, what actions can an individual perform to act on the world to gain information? I previously argued that even for individuals with intellectual disability, systemizing and knowledge seeking are the dominant functions of focused repetitive behaviors and intense interests (12, 13). Lining up objects allows variations in their size and shape (and other properties) to be maximally visible. Piaget (51) referred to lining up objects by height as seriation and considered this ability to mark a major milestone in cognitive development.

Continually turning an object around, while feeling and viewing it from diverse angles, creates minor variations, allowing scrutiny and system-building about the physical parameters of the object. Staring at light through one’s fingertips while varying fingertip openings allows one to gain information about the nature of light (as the plausibly autistic Sir Isaac Newton did when viewing light beams through slits of light such as a barn door crack; 52). Repetitive stair climbing may allow an information-seeking brain to extract regularities from an achievement of human engineering (i.e., stairs; see 53, for a comprehensive analysis of the ingenuity of the staircase). Indeed, I proposed that autistic children’s repetitive behaviors are often centered around the examination of human engineering triumphs (12).

The actor’s own control of the ‘experiment’ is what allows the complexity of information to match the actor’s cognitive abilities. Typically developing infants and children also repeatedly manipulate objects and examine their own fingers to learn, but cease repeating simple motor routines when their cognition is sufficient to allow more complex world exploration. What strikes onlookers as odd and difficult to explain is when repetitive object manipulations continue beyond the toddler years. Reduced social ability then means autistic individuals with intellectual disability are insensitive to social norms and will insist on pursuing ‘experiments to see’ even in the face of distress to caregivers.

#### 4.2.3 Knowledge-seeking has distinct characteristics for different neurotypes

Autism researchers have resisted viewing autistic intense interests as primarily about knowledge seeking even for individuals with normal or high IQ. I propose that a key reason for this is a variant of the double empathy problem (54). NT researchers may be constrained by their own experience of knowledge seeking. Below, I describe three aspects of this.

(1) Learning can be its own reward. For NTs, when knowledge seeking has the specificity observed in autistic interests, this usually includes external goals, which can be multilayered and complex, such as succeeding in school or in one’s workplace or winning status and acclaim (29, 30). Great mental effort is rarely expended solely for the inherent reward of learning. I propose this is part of the reason NT researchers have been stumped in identifying the function of specific interests (as in my prior citation of 17, p. 1797). But fMRI studies have demonstrated that, for autistic adults, mesolimbic reward pathways are most strongly activated by viewing images of their intense interests, with reward pathways hypoactive for social rewards and money (45–48). For the autistic neurotype, the rewards of domain exploration are fierce, and may be stronger than is comfortable for NT researchers to contemplate.

(2) Released from social and external goals, autistic interests can be selected to optimize knowledge gain. This can result in topics alien to mainstream social currency (8, 55). Neurotypical socializing relies heavily on person-centric emotional sharing and strict adherence to age-appropriate social norms (1). This means that neurotypical individuals are primarily interested in broad topics like music, sports, and entertainment (23, 56). Broad topics possess higher social utility because they are statistically more likely to align with a peer’s existing interests. Conversely, highly specialized intellectual topics typically offer lower social utility in mainstream contexts, as the depth of knowledge required for engagement can be a barrier to forming a connection. A useful social strategy is to gravitate toward interests that are plausible venues for daily socializing.

Autistic individuals have more freedom to select topics that optimize the intrinsic rewards of knowledge acquisition and domain exploration. But from the perspective of NT observers, the sheer hyperspecificity of these interests violates expected communication scripts, causing neurotypical observers to perceive them as non-normative or socially jarring (1, 17, 55). The non-normative quality of autistic interests means they can’t be easily understood as knowledge seeking, since for the neuromajority, knowledge seeking generally has a larger purpose. Part of the double-empathy disconnect here is that NT hobbies and interests often serve socializing needs (22).

(3) Hyperspecificity is celebrated, not hidden. I propose that autistics are willing to state their interests at what NTs consider a hyperspecific level, because of differences in display rules between autistic communities and NT communities.

Consider the interest *Recipes from the depression era*. This interest seems odd until one thinks through to the likely rationale for such an interest. During the depression era, poverty and unemployment were widespread, and women were in charge of feeding families. How did women manage to prepare meals? This is a question a historian would pose. Indeed, this is a rather integrative interest. This topic unites home economy, food shortages, female labor, and the all-important challenge of surviving under difficult circumstances. Once one’s brain has gone down this intellectual path, it makes one curious about exactly how such women -- perhaps our own forebears -- rose to these challenges. One then wants to dig into recipes from the depression era, which perhaps were initially found in an elderly relative’s storage box. The interest no longer seems odd once a background rationale is presented.

My argument is that interests seem idiosyncratic because of contemporary display rules about how people are supposed to describe their interests to others. The perceived oddness may thus lie not in the focused interest itself, but in the decision to display rather than suppress it.

In discussion forums, autistic individuals describe their interests to each other. They understand that the display rules are relaxed from what would be expected in a neuromajority context (consistent with the double-empathy problem, 54).

#### 4.2.4 Demand characteristics of the researchers’ agenda

Many autism theorists are ignorant of autistic individuals’ knowledge-seeking because they have not observed autistic individuals describing their focused interests to each other. Researchers such as Dachez and Ndobo (9) acknowledge sense-making and mastery but still place it as a secondary function after emphasizing escaping reality and calming down. They justify the primacy of self-regulation with quotes from their interview, as is the norm and required when analyzing interviews. For example, Dachez and Ndobo (9, p. 89) infer from their interview data:

…their special interests reassure them because they enable them to give meaning to the world around them. They explain that they have trouble naturally understanding the world in which they live. Understanding their environment, via the hard sciences, for example, can be a way of overcoming this problem. Moreover, the fact of understanding soothes them. This illustrates how the search for meaning can be closely linked to the management of emotions….

Why did Dachez and Ndobo’s (9) interviewees report soothing and emotion management, while those in the current forum did this rarely (see **Tables 3**, **4**)? Many autistic adults realize that their interests may strike interviewers as odd, as revealed in studies on autistic masking and camouflaging (57–59). To accommodate expectations, they can explain their interests in terms that will make sense to their neurotypical interlocutors. Of course, many autistic individuals do find their interests self-regulating, just as neurotypicals find their hobbies relaxing and soothing, and so they may work this into their explanation. Here we see how analyzing online discussion forums is a complementary method to surveys and interviews, by minimizing the demand characteristics and researchers’ agendas (see **Table 4**; 32).

Note that intense interests did serve knowledge acquisition for Dachez and Ndobo’s (9) interviewees. The authors mentioned this here:

Accumulating knowledge enables them to have points of reference in order to build a coherent vision of their environment. For others, their special interests enable them to have a better understanding of human beings and social relationships. The human being thus becomes a subject of study, through the analysis of psychology, for example.

The posters reported in the current study were open about how curiosity about people’s lives allowed learning about social relations; an interest in true crime mixed the fun of sensationalism with details about human motivational systems.

### 4.3 Implications for diagnosis

A key result of this forum analysis is that the motivation for intense interests is the intrinsic rewards of learning. This motivational orientation is relevant to whether an intense interest is suggestive of an underlying autism phenotype, by considering how the interest is integrated into diverse aspects of the person’s life. Integration is important because many of the interests described here may not meet diagnostic criteria for a restrictive, repetitive behavior. After all, the behaviors are often insufficiently perseverative or impairing (55, 60).

Still, many of the interests reported here are socially jarring due to being hyperspecific, as in *collecting bat figurines; caring for succulents; and being able to identify any pure breed of dog just by a partial view of their body such as the tail.* But these interests are on a continuum with neurotypical collecting (stamps, coins, vintage watches; 61); animal enthusiasts (birding, dog showmanship) and gardening (62). If the systemizing potential of interests and hyperspecificity of interests do not discriminate autistic intense interests from neurotypical hobbies, can intensity be the salient factor? This is unclear. Many neurotypical collectors and animal enthusiasts approach their interest with an intensity that can impair one’s family commitments (recall the popular label, ‘video games widow’; e.g., 63, 64). The disrupting potential of hobbies is also referenced in the title of Gillespie et al.’s (65) *Leisure Studies* article: *If it weren’t for my hobby, I’d have a life: dog sports, serious leisure, and boundary negotiations.*

#### 4.3.1 Motivations for learning varies between autistic and neurotypical neurotypes

I propose that a method for discerning if hyperspecific, academic and/or intellectual interests are suggestive of an underlying autism phenotype is whether the interest is embedded in larger reward systems, beyond the inherent rewards of learning. These reward systems are evident in the motivations underlying hobbyists in the ‘serious leisure’ group (66). Consistent with self-determination theory, these motivations involve diverse goals, most commonly: social engagement, identity, competition, and community commitment. Below I list how these motivations play out in three domains that resemble the academic and intellectual interests reviewed in the current forum analysis.

- Hyperspecific collecting. These are usually well-known domains (not arbitrary collecting) which provide social identity and community with other collectors (61).
- Birding. As birders become more specialized, their motivations shift towards competition, which includes completing a life-list of rare species encountered. Lists are shared and compared with fellow birders (67).
- Dog Sports and Breed Enthusiasts. Enthusiasts operate within a subculture that offers identity and ingroup recognition in return for adopting a strict ‘dog person’ lifestyle, characterized by insider knowledge of breed standards and subcultural jargon (65).

#### 4.3.2 A specific hypothesis about motivations for learning

Future research can test this hypothesis: The autism phenotype is most strongly suggested if the primary reward is learning (or exploring), with weaker roles for social engagement, identity, competition, and community commitment. Diagnosis is complicated because many autistic adults may embrace the communities that organize around these hyperspecific hobbies (21). ‘Serious leisure’ activities (66) attract autistic people and those with autistic traits (68). Autistic adults with typical and high intellectual ability generally recognize the value of these communities and may become valued members, also complicating diagnosis (69, 70).

We can try out this method by applying it to a post which concerned diagnosis: a woman noted that her interest in interpersonal relations was the reason her mother had dismissed autism as a possibility, due to stereotypes that autistics are interested in mechanical systems. **Table 2** shows that psychology was one of the most frequently mentioned interests (see Section 3.1.1), but it was also one that had an explicit goal: to understand people. Has this example just disconfirmed my method for using motivation and goals to inform diagnosis?

One nuance here is that the explanation in this example flags weak social skills, which is consistent with the autism phenotype. Also, this poster wanted to learn about interpersonal relationships. The focus is on *learning*, not engaging socially, gaining an identity, achieving via competition, or committing to a subculture.

### 4.4 Implications for clinical support

Clinicians’ most pressing challenge is working with autistic children and adults who have intellectual disabilities, not the autistic adults whose forum posts were analyzed here. But if clinicians entertain the idea that repetitive motor behaviors are knowledge-seeking, they can direct those ‘experiments to see.’ For example, if a client is repeatedly looking at light through their fingers, a clinician can introduce prisms, colored lenses, or shadow-play to feed that cognitive curiosity in a richer way. This is similar to strategies in Floor Time (71), where clinicians recognize sensation seeking and provide an enriched sensory diet. My suggestion is to determine whether knowledge seeking exists in addition to sensation seeking. If so, conceptualize goals in ways that guide their client to more knowledge, which could influence behavior via endogenous brain rewards (see also, 6).

#### 4.4.1 Support for autistic clients with typical intellectual ability

Recommendations have circulated for years for clinicians to validate their clients’ special interests as a positive aspect of their identity and a source of well-being, rather than pathologizing them as behavioral deficits (6, 14, 21, 72). I add to this the following points, which are less often discussed.

- Encourage autistic individuals to read autistic forums to help them process self-acceptance of having interests that are deemed by the larger society as too intense or eccentric (17).
- Encourage autistic individuals to discover how their interests are on a continuum with the knowledge-seeking of scientists and scholars. This can reduce the standard deficit view of autism.
- Autistic youth can be guided towards the rewards of extending their interests beyond focused domain exploration, into applied arenas. These have been frequently discussed, but are challenging to implement. Examples include finding friends with their interests, creating a club for their interests at school, or taking up a relevant policy issue in their community (17, 35, 73).

### 4.5 Implications for self-understanding

The current forum analysis contributes to the larger field of the science of human curiosity (24, 25, 28–30, 74). People are often curious about why they are attracted to certain topics and why their passions may wax, wane, and change over time (75). Learning more about the reasons for interests is especially important for autistic individuals, given that their interests frequently differ from others’ expectations. This curiosity to understand one’s specialized interests may be the reason so many forums exist where autistic people ask each other to share their interests.

Difficulty finding people who share an idiosyncratic interest can lead to frustration, as in the poster who wrote, “All I want is someone … I can talk about my interests with all day…” It can help to learn that many others have similarly intense and specialized interests. Clinicians can also emphasize that cognitive styles of systemizing and desire for comprehensive domain exploration can be viewed as positive aspects of autism spectrum conditions (7, 49, 76).

### 4.6 Implications for leveraging interests for careers

#### 4.6.1 A good fit – autistics’ intellectual interests and academia?

A growing literature exists on how autistic adults can develop their interests into careers (1, 3, 9, 77). I will set aside careers in general and focus on how intense interests can be leveraged into a career as a scientist or college professor. The structured, detail-oriented nature of academia has been reported to be a sanctuary for individuals with intense cognitive focus (78). Administering the autism quotient survey to a wide variety of respondents revealed elevated autistic traits among scientists and mathematicians compared to the general population (68).

Memoirs and reports exist on the challenges autistic individuals experience when pursuing academic careers (79–83). These include the exhaustion of masking (82), navigating hierarchy and the “hidden curriculum,” a nuanced topic that includes decoding implicit social rules (79, 84, 85). Some memoirs imply that drawing on one’s curiosity, love of learning, and intellectual interests will be the easy part of academia for autistic individuals, with the masking, hierarchy and implicit social rules being the challenging part. I do not dispute these challenges, but to add value to the conversation, I set aside masking, hierarchy and implicit social rules and address what is reputed to be the easy part: leveraging one’s intense intellectual interests for a career in academia.

#### 4.6.2 Motivation for special interests

Intensity of interests and systematization as a motivating drive most likely vary dimensionally throughout autistic and neurotypical populations (86). But for narrative ease, I describe the motivations for interests between autistic individuals and career academics in somewhat categorical terms.

While career academics relish the rewards of domain exploration, they are additionally motivated by specific career goals, and know how to leverage academic discoveries to build their brand (87). Numerous articles now document the tension between satisfying one’s curiosity via research vs. meeting the demands of institutional academia (88). Career academics succeed by keen message discipline. They keep their eye on the prize of career advancement (87).

Let me note something a bit paradoxical. I’ve argued that autistics’ interests are the opposite of restricted and circumscribed; that is, autistic interests are wide-ranging, covering numerous topics. Autistics may have too many interests to easily succeed in academia. Academia requires decades-long fixation on a restricted set of narrow interests, thoroughly explored (89).

Autistic adults typically have many interests, as reported in the forum posts (also 8). Cumin et al. (90, p. 61) described exhaustive exploration in brusque terms, such as this quote from one of their interviewed clinicians:

[Clinician P02] described autistic deep interests as ego-syntonic, exhaustive, but also cyclical: “It’s not so much that they lose interest, but they move on to something else once they realize they have drawn all possible functional benefits out of the interest.”

Cumin et al. (90, p. 61) acknowledged that exploring domains serves intellectual purposes, e.g.: Clinicians also agreed that autistic women often had “useful” deep interests, which they could apply to facilitate social interaction. But Cumin et al. (90) stopped short of using terms like curiosity and knowledge-seeking.

#### 4.6.3 Career academics’ magic bullet: keen message discipline

Academics frequently begin their careers with wide-ranging curiosity, and may continue to pursue these wide-ranging interests as hobbies. Misalignment between curiosity and careerism contributes to driving doctoral dropout rates to between 40% and 50% across most disciplines (91). Those who succeed in academia often do so via strict message discipline. They stay focused on becoming experts in a small number of areas and being strategic in leveraging their expertise for career goals (92, 93).

Achieving message discipline is difficult (91–93). Aspiring academics of all neurotypes may flounder as new topics of interest arise, pulling them in a different direction (94). They may become bored with a topic or feel they have explored it sufficiently and thus seek something new. To stay on track, these young scholars typically require guidance from mentors, but many leave academia due to these and related difficulties (91, 93). Recall the quote from Cumin et al., regarding autistic women’s need to move on to a new topic once they have exhausted the benefits of exploring the prior topic. In contrast, successful scientists and scholars learn strategies to almost indefinitely extend their exploration of a focused domain (95). The primary method is to gain research funds to push the boundaries of their domain (moving deeper within the same domain). A second strategy is to stay in that domain, but move up a level to be an administrator of knowledge, such as mentoring junior scientists, writing books, or developing relevant public policy (96).

Many autistic individuals are already successful in academia, although they may not be publicly labeled as autistic (84, 85). Some autistic individuals are thus already able to figure out the strategies to incorporate their interests into academic careers. Some have proposed changing academia to be more flexible to accommodate autistic individuals’ cognitive style (e.g., 80, 84, 85). Future research can investigate whether autistic academics are helped by following the strategies of neurotypical academics, which include using interests as a means to an end.

## 5 Limitations and future directions

### 5.1 An educated, introspective sample

Participating in online discussion forums likely attracts individuals who have an intellectual orientation, need for cognition (which facilitates wide-ranging interests) and enjoyment of learning for its own sake, which are the key findings of this analysis. The findings of this analysis are thus likely most generalizable to the subgroup of autistic adults who have these characteristics. However, the knowledge-seeking and curiosity-driven learning observed in these forum posts overlaps with long-standing observations about autism. Autistic individuals are known for scientific pursuits (Silberman, 2015), systemizing drives (5, 33, 49) and enhanced logical reasoning ability (97). However, one can additionally question generalizability to cultures outside the educated English-speaking world, given that educated, English-speaking cultures are already global outliers for analytical cognitive style (98).

### 5.2 More posts from women than men

A primary limitation of the current study is that the gender of posters was not controlled. The majority of posts were drawn from female-focused digital spaces (e.g., r/Aspergirls, r/AutismInWomen; see **Table 1**). Consequently, the finding that knowledge-seeking and systemic learning serve as the primary drivers of intense interests is most accurately situated within the literature on the female autism phenotype. Readers may have already observed many female-oriented topics (notably, *cooking, fashion, knit crafts, the beauty industry, and true crime;* the latter of which heavily draws a female demographic; 99). This presents a unique theoretical opportunity. It has become increasingly well-known that autistic women (and especially girls), more than autistic males, gravitate toward culturally normative interests (100, 101). This masks their diagnostic status from clinicians who expect male-associated topics like mechanical systems or train schedules (59, 102). The important inference is that, even when the content of these interests aligns with mainstream female subcultures, the underlying cognitive drive remains knowledge-oriented.

Outside of the stereotypically female topics listed above, the content analysis revealed mostly gender neutral interests. The broad organization of the content analysis in **Table 2** is thus expected to be preserved in an analysis of autistic male posters. Furthermore, one would expect autistic males to resemble autistic females in being wide-ranging and intellectually oriented.

Gender-comparative research remains a vital next step. Future studies can deliberately partition data by leveraging platforms where gender-identified user profiles are standard practice, such as *wrongplanet.org* or by searching for Reddit posts of users with biographical tags.

### 5.3 Future research: more analysis of goals

A future analysis can more closely examine motivations and purpose. Consider the post *Every time I grow/make something … I feel like I’m hacking “the system.”* This focus on achievement moves beyond learning, but the main reward may continue to be learning, as in learning to hack the system, learning to be efficient. This is still not the purpose one sees in the sociology of leisure studies (socializing, identity, competition, community), but is recognizable as a common human goal that is at least somewhat distinct from systemizing, learning and knowledge-seeking.

## 6 Conclusions

Prior researchers have noted that special interests should be more positively regarded, rather than being viewed as burdensome and maladaptive (7, 14, 21, 72). This analysis takes that insight to a new level. The intellectual appetite of autistic people has not been appropriately recognized. More than merely having useful talents in circumscribed areas, the interest in constantly learning new topics opens the door for autistic people to make intellectual contributions to society. Technological changes will mandate life-long learning, a trait endorsed by many of the forum posters analyzed here.

## Data Availability

The raw data supporting the conclusions of this article will be made available by the author, without undue reservation.
